# Obtaining Patient-Reported Outcome Data via a Home Patient Monitoring App: Development, Implementation, and Validation of Novel Interface Terminology

**DOI:** 10.2196/65504

**Published:** 2025-12-11

**Authors:** Lucia Sacchi, Giordano Lanzola, Silvana Quaglini, Nicole Veggiotti, Silvia Panzarasa, Valentina Tibollo, Matteo Terzaghi, Itske Fraterman, Savannah Glaser, Manuel Ottaviano, Vadzim Khadakou, Vitali Hisko, Mor Peleg, Sofie Wilgenhof, Henk Mallo, Alexandra Kogan, David Glasspool, Stephanie Medlock, Laura Del Campo, Matteo Gabetta, Mimma Rizzo, Laura Deborah Locati, Paola Gabanelli, Sara Demurtas, Andrea Premoli, Szymon Wilk

**Affiliations:** 1 Department of Electrical, Computer and Biomedical Engineering, University of Pavia, Via Ferrata 5, Pavia, 27100, Italy, +39 0382 985218; 2 Medical Informatics and Artificial Intelligence Laboratory (LIM-IA), IRCCS Istituti Clinici Maugeri, Pavia, Italy; 3 The Netherlands Cancer Institute, Amsterdam, The Netherlands; 4 Department of Medical Oncology, University Medical Center Groningen, Groningen, The Netherlands; 5 Amsterdam UMC - University of Amsterdam, Medical Informatics, Amsterdam, The Netherlands; 6 Amsterdam Public Health, Methodology & Digital Health, Amsterdam, The Netherlands; 7 Life Supporting Technologies, ETSI Telecomunicación, Universidad Politécnica de Madrid, Madrid, Spain; 8 Bitsens JSC, Vilnius, Lithuania; 9 Department of Information Systems, University of Haifa, Haifa, Israel; 10 Deontics Ltd, London, United Kingdom; 11 Amsterdam Public Health, Methodology & Aging & Later Life, Amsterdam, The Netherlands; 12 Associazione Italiana Malati di Cancro, Parenti e Amici (AIMAC), Roma, Italy; 13 Biomeris Srl, Pavia, Italy; 14 Medical Oncology Unit, Azienda Ospedaliera Universitaria Consorziale Policlinico di Bari, Bari, Italy; 15 Department of Internal Medicine and Medical Therapeutics, University of Pavia, Pavia, Italy; 16 Medical Oncology Unit, IRCCS Istituti Clinici Maugeri, Pavia, Italy; 17 Psychology Unit, IRCCS Istituti Clinici Maugeri, Pavia, Italy; 18 Institute of Computing Science, Poznan University of Technology, Poznan, Poland

**Keywords:** adverse effects, cancer, clinical decision support system, decision-making, home patient, mHealth, mobile app, patient-reported, patient-reported outcome, patient with cancer, quality of life, self-reporting, symptom assessment

## Abstract

**Background:**

Adverse events (AEs) related to cancer treatment represent a valuable source of information that can be used to adjust therapy for individual patients. The National Institutes of Health developed the Common Terminology Criteria for Adverse Events (CTCAE), a comprehensive standardized terminology for health care providers to consistently report AEs during patient visits. Mobile health technologies, in principle, also allow AEs to be self-reported by patients in between visits; however, the terminology poses challenges for them, both in selecting the correct symptom to report and in rating its severity. The National Institutes of Health developed the Patient-Reported Outcomes–CTACE as the patient-oriented *companion* of the CTCAE. However, it shows some weaknesses in completeness and precision when used for continuous home patient monitoring and for decision support.

**Objective:**

The aim of this paper is to propose a new terminology for reporting AEs that is easy for patients to use while also being clinically meaningful for health care providers and easily exploitable by decision support systems. Moreover, we aim to demonstrate its implementation and validation within the CAPABLE (Cancer Patients Better Life Experience) EU project.

**Methods:**

The development of the new terminology starts from the CTCAE, which includes a comprehensive list of signs and symptoms along with guidance for accurately grading their severity. Through a multistep, participatory approach involving both patients and health care providers, we reduced and adapted the AE list for patient-oriented apps. During the CAPABLE project, the proposed terminology was integrated into a mobile app and evaluated within a clinical pilot study involving 86 patients who were monitored through the app for at least 6 months, and a control cohort of 133 patients monitored using standard care practices.

**Results:**

The final terminology includes 124 AEs, 49 expressed as “present or absent,” and 77 associated with 4 description levels. A mapping between the description levels and the original CTCAE grades enables running the decision support system embedded in the CAPABLE app. The pilot study demonstrated that the majority of the patients used the symptoms reporting functionality, sharing also 24 unique AEs that are not present in the Patient-Reported Outcomes–CTCAE. Symptoms reported using the proposed terminology allowed the enactment of the clinical practice guidelines included in the CAPABLE decision support tool, triggering 11 distinct recommendations.

**Conclusions:**

The results obtained from the clinical study support our claim regarding the need for a novel terminology for the self-reporting of AEs, characterized by ease of use, completeness, and clinical meaningfulness. Finally, by mapping our terminology to the CTCAE, we demonstrated that it is possible to exploit self-reported data to trigger decision support rules consistent with clinical practice guidelines.

## Introduction

During patients’ follow-up, a complete and accurate recording of symptoms helps health care providers to make correct diagnoses and improve their decision-making. However, the collection of symptoms is almost exclusively done during patient visits. This makes it less accurate and complete, due to recall bias on the patient’s side, and underreporting on the clinicians’ side, caused by short visit duration, failure to ask questions systematically, and attribution bias [1,2]. Patient-reported outcomes (PROs) can mitigate these issues. They are defined as “‘any report of the status of a patient’s health condition that comes directly from the patient without interpretation of the patient’s response by a clinician or anyone else.” This definition comes with the claim by the Food and Drug Administration of their usefulness in assessing drug efficacy in clinical trials [3]. More recently, another regulatory body, the European Medicines Agency, launched the “Regulatory Science Strategy to 2025,” one of the key goals of which is “advancing patient-centered access to medicines in partnership with healthcare systems.” This includes integrating PROs and patients’ preferences with additional data collected during the patient’s clinical path to evaluate the drug risk profile [4]. Therefore, while the collection of PROs also presents challenges [5], it holds significant promise, and not only in the drug surveillance area [6].

PROs gained additional momentum with the widespread adoption of mobile health (mHealth) apps. As a matter of fact, several implementations of the so-called electronic patient-reported outcomes measures (ePROMs) have been developed [7]. The benefits of ePROMs include improved symptom control, physical function, quality of life (QoL), adherence to treatment, reduction in hospital admissions, improved survival, and communication between patients and health care providers [8-15].

Which terminologies and data collection approaches are suitable for ePROMs depend on the purpose of their use. In one of their seminal papers, Basch et al [16] describe a web application designed for patients with gynecological malignancies, who could access it on the day of their appointment using computer kiosks in hospital waiting rooms or their personal devices. Symptoms were collected through a questionnaire with 7 items adapted from the National Cancer Institute’s (NCI) Common Terminology Criteria for Adverse Events (CTCAE) [17] plus a performance status item. Free text was available for entering additional information. Since then, several systems have been developed. PatientViewpoint is a web-based system, relying on questionnaires that patients fill out at regular time intervals upon system reminders [18]. Questionnaires, which can be selected according to patients’ condition, were built on the PROMIS and Functional Assessment of Chronic Illness Therapy system [19]. STAR (Symptom Tracking and Reporting) is a web-based interface for patients with a high symptom burden, whose effectiveness was demonstrated in a randomized clinical trial on patients with advanced solid tumors [15]. It includes questions from the CTCAE, adapted for patient use, and pertaining to 12 common symptoms experienced during chemotherapy, graded on a 5-point scale (from 0 to 4) of increasing disability. Basch et al [20] describe PRO-Core, which includes a survey with questions from the NCI’s Patient-Reported Outcomes–Common Terminology Criteria for Adverse Events (PRO-CTCAE) validated item library [21], selected based on prevalence across different types of advanced cancers, as well as questions about oral drug intake and performance status. Velikova et al [22] describe eRAPID, a system relying on internet-based questionnaires and managing the connection with the hospital electronic health record. The questionnaire items were identified according to different cancer types, patient interviews, and systematic reviews of PRO reporting for those groups.

A common feature of the systems described above is their use of questionnaires that reference predefined time periods (eg, the past week) for data collection. Moreover, the questionnaires contain only a subset of all the possible adverse events (AEs), tailored to the addressed population and context.

As a matter of fact, the real-time, longitudinal collection of symptom data directly from patients, up to the resolution of their condition, is inherently complex [23,24]. One of the important challenges is precisely the definition of a suitable terminology to be used at the moment of symptom reporting. To address this challenge, in this paper, we aim at developing a terminology that, differently from the already available literature, is (1) general enough to be suitable for different patient populations; (2) appropriate for monitoring home patients, that is, patients receiving treatment at home, possibly having a home caregiver, and potentially undergoing extended follow-up periods; and (3) exploitable by a system that provides real-time support for AE management.

The terminology that we describe was developed as part of the European Horizon 2020 project CAPABLE (Cancer Patients Better Life Experience). Despite CAPABLE focusing on home patients with cancer, aiming to improve their QoL, the produced terminology is broadly applicable across patient populations.

The CAPABLE system consists of 2 front-end components and a back-end component [19]. The 2 front-end components are a mobile app for patients and a web-based dashboard for the health care providers.

The app supports patients in managing their condition. It includes several functionalities (educational material, mental well-being activities, suggestions for physical exercise, etc), but since a major issue for oncological patients is the occurrence of treatment-related AEs, its main goal is to provide easy and accurate reporting of AEs, in terms of signs and symptoms that patients may experience. It includes a symptom follow-up strategy to enhance monitoring compliance. Upon patients’ consent, the app may also be used by home caregivers. This allows reporting those symptoms that, in certain conditions, a patient would not be able to report, for example, delirium. The dashboard is a web application through which health care providers can monitor patients’ data remotely.

The back-end component [25] implements the CAPABLE decision support system (DSS) [26], which includes (1) a *virtual coach*, aimed at providing feedback to patients and their home caregivers through the app, and (2) a *physician DSS,* implemented as a set of computer-interpretable clinical guidelines providing evidence-based recommendations for doctors about the management of the reported AEs [27-31].

The objective of this paper is to introduce the AE-reporting terminology developed in the CAPABLE project. As mentioned, different from the terminologies already presented in the literature, we herein propose a highly flexible ePROs collection system, which allows patients to choose from an exhaustive list of signs and symptoms, enter data at any time, and manage the follow-up of the reported symptoms according to clinical practice guidelines. Finally, we present the evaluation of the terminology conducted as part of a clinical study involving patients who either used the app or received traditional follow-up care.

## Methods

### Overview

The process of defining a novel terminology began with an analysis of the 2 main existing AE terminologies, CTCAE and PRO-CTCAE, to identify their respective strengths and weaknesses. Insights from this analysis informed the strategy for creating the new terminology. In this section, we describe the whole process in detail.

### Analysis of CTCAE and PRO-CTCAE

The CTCAE version 5 is a terminology developed by the NCI that allows standardized reporting of abnormal clinical findings, along with their grade of severity, which may range from grade 1 to grade 5. As shown in Table 1, grading may be based on the impact of the symptom on activities of daily living (ADLs, first row), or it may consider (also) objective descriptions of the symptom that represent increasing severity (eg, second row, where objectivity is based on the increased number of stools/day).

**Table 1.** Examples of the Common Terminology Criteria for Adverse Events (CTCAE) terminology, showing name, definition, and grading of 3 adverse events (source: National Cancer Institute [32]).

CTCAE term	Grade 1	Grade 2	Grade 3	Grade 4	Grade 5
Ear pain^a^	Mild pain	Moderate pain; limiting instrumental ADLs^b^	Severe pain; limiting self-care ADLs	—^c^	—
Diarrhea^d^	Increase of <4 stools per day over baseline; mild increase in ostomy output compared to baseline	Increase of 4‐6 stools per day over baseline; moderate increase in ostomy output compared to baseline	Increase of ≥7 stools per day over baseline; incontinence; hospitalization indicated; severe increase in ostomy output compared to baseline; limiting self-care ADLs	Life-threatening consequences; urgent intervention indicated	Death
Anemia^e^	Hemoglobin 10 g/dL—LLN^f^	Hemoglobin 8‐10 g/dL	Hemoglobin <8 g/dL; transfusion indicated	Life-threatening consequences; urgent intervention indicated	Death

a A disorder characterized by a sensation of marked discomfort in the ear.

b ADL: activity of daily living.

c Not available (CTCAE defines the severity for this adverse event only in the range 1-3).

d A disorder characterized by frequent and watery bowel movements.

e A disorder characterized by a reduction in the amount of hemoglobin in 100 mL of blood. Signs and symptoms of anemia may include pallor of the skin and mucous membranes, shortness of breath, palpitations of the heart, soft systolic murmurs, lethargy, and fatigability.

f LLN: lower limit normal.

CTCAE is a de facto standard adopted by several scientific societies and in particular by the European Society of Medical Oncology, whose guidelines have been implemented in the CAPABLE DSS [27-31]. For example, the guideline on immune-related AEs [31] explicitly refers to CTCAE grades in its statements about the discontinuation of immune checkpoint inhibitor (ICI) therapy in case of immune-related neuropathy:

(1) for grade 1 symptoms, ICI treatment can be continued and the patient is monitored for deterioration; (2) for grade 2 symptoms, ICI treatment should be interrupted and oral or i.v. (methyl)prednisolone is initiated; and (3) For grade 3 and 4 symptoms, more intensive immune modulation may be required in addition to corticosteroids [...]

The CTCAE includes more than 800 AEs, and it was designed to be used by health care providers; thus, it cannot be directly exploited as an interface terminology for a patient’s app. One main reason is that several AEs are in fact medical diagnoses that patients or home caregivers cannot detect on their own because they require laboratory tests (see, eg, footnote “d” of Table 1 where anemia is defined).

Another challenge is the reliance on medically oriented technical language. For example, rash is described as “Macules/papules covering x% of the body surface…”, where x determines the severity grade. Such terminology is clearly difficult for lay users to interpret.

The NCI also developed the PRO-CTCAE Measurement System, which is intended as the CTCAE companion terminology for patients [21]. As shown in Textbox 1, PRO-CTCAE allows reporting AEs either as present or absent or by specifying one or more of the following attributes: *frequency*, *severity*, *interference with daily activities*, and *amount*.

Textbox 1. **Patient-Reported Outcomes–Common Terminology Criteria for Adverse Events approach to adverse event reporting.**

**Frequency**

- In the last 7 days, how often did you have…?
  - Never
  - Rarely
  - Occasionally
  - Frequently
  - Almost constantly

**Severity**

- In the last 7 days, what was the severity of your…at its worst?
  - None
  - Mild
  - Moderate
  - Severe
  - Very severe

**Interference**

- In the last 7 days, how much did…interfere with your usual or daily activities?
  - Not at all
  - A little bit
  - Somewhat
  - Quite a bit
  - Very much

**Amount**

- In the last 7 days, did you have any…?
  - Not at all
  - A little bit
  - Somewhat
  - Quite a bit
  - Very much

**Present or absent**

- In the last 7 days, did you have any…?
  - No
  - Yes

PRO-CTCAE has been employed in several studies to collect AEs in patients with cancer and has been shown to enhance the quality of symptom reporting compared with standard reporting during routine visits [33]. It has also been associated with increased patient empowerment and improved QoL [34].

However, the PRO-CTCAE alone was not sufficient for our needs for the following reasons:

1. It includes only 80 AEs, while there are many more symptoms or signs included in the CTCAE, which are potentially detectable by patients or their home caregivers, as outlined by our analyses conducted with health care providers and patients.
2. The patient is asked to report on symptoms experienced *in the past 7 days*. First, this is not suitable for symptoms that need to be managed as soon as they arise, without having to wait for the next reporting window. Second, this might force patients to fill in extra daily diaries to avoid forgetting some short-duration symptoms after a couple of days [34].
3. Symptom severity is only subjectively assessed, using a Likert scale measuring the impact on daily life activities, while it would be more appropriate to use (also) objective features, which could help health care providers to better understand the patient’s condition.
4. As a consequence of (3), some of the PRO-CTCAE entries were hardly mappable to the CTCAE, even if efforts in this sense are ongoing [35]. Thus, in a system where patients enter data using the PRO-CTCAE and health care providers enter data using the CTCAE, there is an issue for data integration and their use in statistical analyses or DSSs (as mentioned, clinical practice guidelines refer to the CTCAE in their recommendations).
5. Different from the CTCAE, the PRO-CTCAE does not include any suggestions for symptom management. For example, in Table 1, we can see “hospitalization indicated” for Grade 3 diarrhea, which could be translated for a patient as “go to the emergency room” or “contact your physician.”

In sum, the CTCAE, being developed for health care providers, is not appropriate for direct use by patients, whereas the PRO-CTCAE, despite being a validated and appropriate tool for a qualitative assessment of a patient’s condition, has some drawbacks if included in a patient monitoring system that requires a complete, quantitative, precise, and timely assessment.

### Development of the Novel CAPABLE Terminology

**Figure 1.**
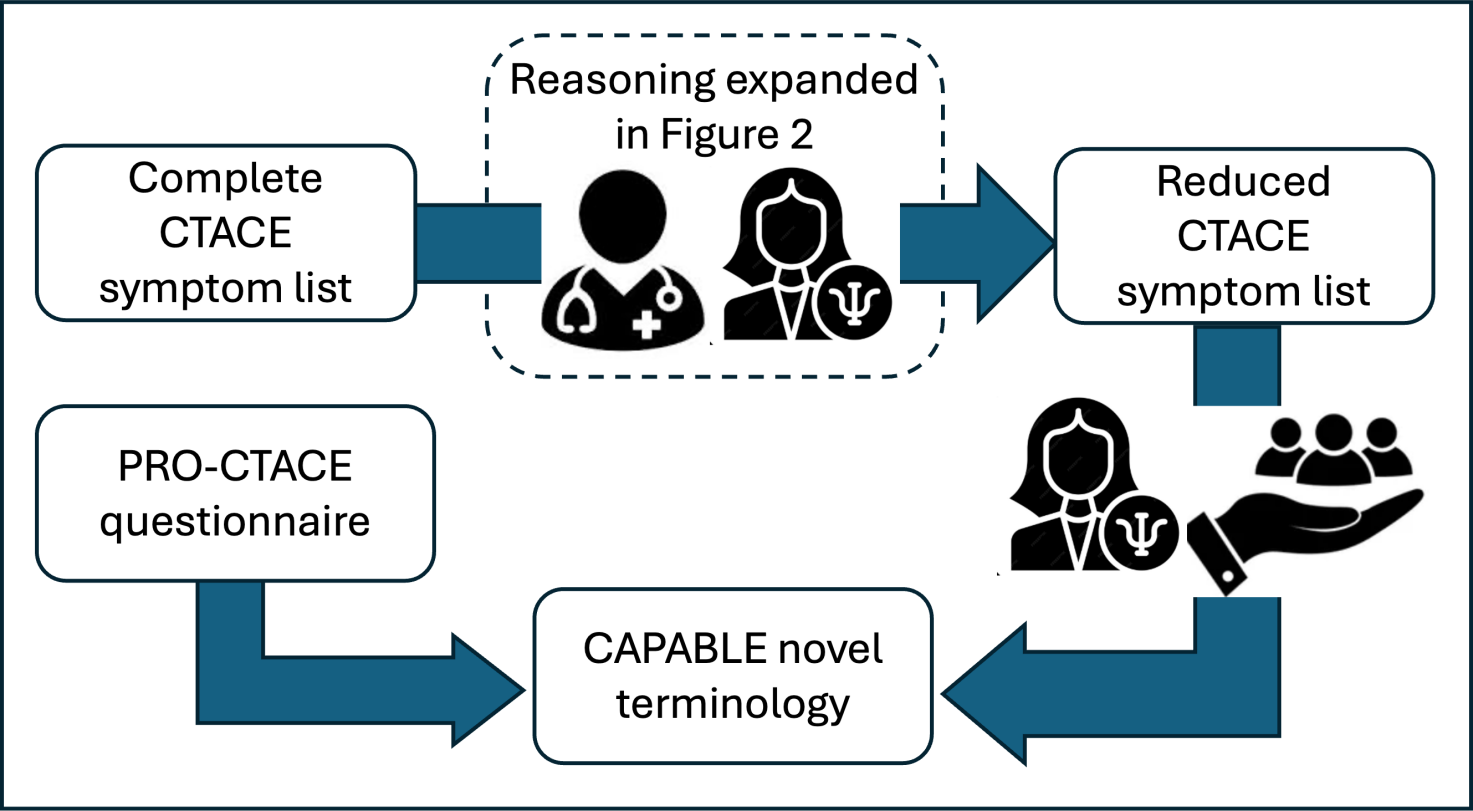
Overall framework of the process used to develop the new terminology. CAPABLE: Cancer Patients Better Life Experience; CTACE: Common Terminology Criteria for Adverse Events; PRO-CTACE: Patient-Reported Outcome–CTACE.

To overcome the mentioned limitations of the existing terminologies, we decided to start from the CTCAE and review it in depth. Figure 1 shows the high-level process schema. Oncologists and psychologists worked together to filter the CTCAE AEs list, based on the estimated patients’ ability to recognize symptoms and on other features illustrated below, while psychologists and patients collaborated to replace complex terms with more accessible ones. When suitable, PRO-CTCAE terms were adopted.

More precisely, we went through the full list of AEs and performed the following steps:

1. Remove AEs that are not detectable by a patient or his or her home caregiver (eg, anemia).
2. Manage AEs, such as anxiety, depression, and sexual life, with dedicated and validated questionnaires, as suggested by our medical experts based on the observation that CTCAE terms for those conditions are not sufficiently detailed for monitoring purposes. These AEs are thus excluded from the terminology.
3. Remove AEs related to some vital signs, for example, weight gain or loss, or hypertension, which can be easily inferred from the multiple measures of body weight and blood pressure that patients periodically report in the app.
4. Remove AEs that are specializations of other AEs already informative enough for the oncologists (eg, hoarseness can be removed as it is covered by the general term voice change).

Once the reduced list was obtained, we performed the following additional steps:

1. Remove grade descriptions referring to death and life-threatening situations that are outside the CAPABLE scope and could be alarming for patients. This step was suggested by the psycho-oncologists working on the project.
2. Remove from the grade descriptions any statements referring to medical interventions, as these serve as guidance for clinicians rather than for patients.
3. Rephrase the description of AE names and grades using words that are easily understandable for patients. When possible, we exploited the PRO-CTCAE description. For some AEs, a CTCAE grade was split into 2 levels to allow patients to better describe their situation. For example, in the CTCAE, cough is described by 3 grades: mild, moderate, and severe symptoms. In our terminology, we provide 4 possible descriptions, splitting the *severe* level into (1) severe cough that interferes quite a bit with my usual or daily activities and (2) very severe cough that interferes with my daily self-care activities.
4. Employ a principle of the lowest risk of underestimation: if the distinction between grade descriptions of a symptom might not be clear for a patient, the symptom will be mapped to a higher severity level. For example, the CTCAE assesses the severity of pruritus based on whether the symptom is localized or generalized. This may be difficult for a patient to evaluate, so it was omitted from the descriptions, which measure the impact on ADLs instead. Thus, the report of a highly impacting itching would be mapped to CTCAE grade 2, instead of grade 1, even if localized. A physician can then assess the symptom and assign the correct CTCAE grade.

The last 2 points were carried out in collaboration with patients belonging to the Associazione Italiana Malati di Cancro patients’ association (in Italy) and 2 expert nurses (in the Netherlands) to ensure the understandability of the new proposed phrasing. The terminology was developed in English, Italian, and Dutch, using the localized version of the available CTCAE and PRO-CTCAE terminologies as a basis.

### Evaluation of the Proposed Terminology

CAPABLE was tested within a clinical pilot study that took place in 3 clinical centers, 2 in Italy and 1 in the Netherlands, from May 2023 to January 2024 (ClinicalTrials.gov NCT06161233 and NCT05827289) [36]. The system was used by 86 patients overall. In total, 2 main cancer types were addressed, metastatic renal cell carcinoma (mRCC; 30 patients: 20 males and 10 females—with a median age at enrollment of 62 y) and melanoma (31 patients: 12 males and 19 females—with a median age at enrollment of 65 y; one of these patients was lost at follow-up), and breast, head and neck, lung, stomach, ovary, and thyroid cancer were considered (26 patients overall: 5 males and 21 females, with a median age at enrollment of 50.4 y). An additional 133 patients were enrolled from May 2021 to March 2023 into a control cohort that did not use the CAPABLE app. The control cohort consisted of 77 patients with mRCC, 56 males and 21 females, with a median age at enrollment of 60 years, and 56 patients with melanoma, 22 males and 34 females, with a median age of 64 years.

### Ethical Considerations

The studies were approved by the Ethical Committees of the clinical centers as follows: EC #2546 and EC #2741 ICS Maugeri—Pavia, EC #7558 Azienda Ospedaliero-Universitaria “Consorziale Policlinio”—Bari, and EC #22‐981/NL81970.000.22, NedMec EC Amsterdam. Informed consent was obtained from all the patients participating in the studies. All patients also signed a data-processing consent form, and a Data Protection Impact Assessment was prepared and approved by each clinical center. The data used for the analyses presented in this paper were deidentified before being processed by the authors responsible for the analyses, who signed a data-processing agreement with the clinical centers. No compensation was provided to the study participants.

In addition to the clinical study, which allowed the collection of clinical data for a quantitative assessment of the intervention efficacy (the primary outcome of CAPABLE was QoL), a focus group was held near the conclusion of the study itself to gather qualitative data from the user experience. Moreover, at the end of the study, questionnaires on the ease and usefulness of the system were collected from patients and health care professionals. These questionnaires included 1 item for each functionality of the CAPABLE systems, including the symptom reporting, to help understand how patients and health care providers perceived the functionality, also in comparison with the others available.

## Results

### The Developed CAPABLE Terminology

**Figure 2.**
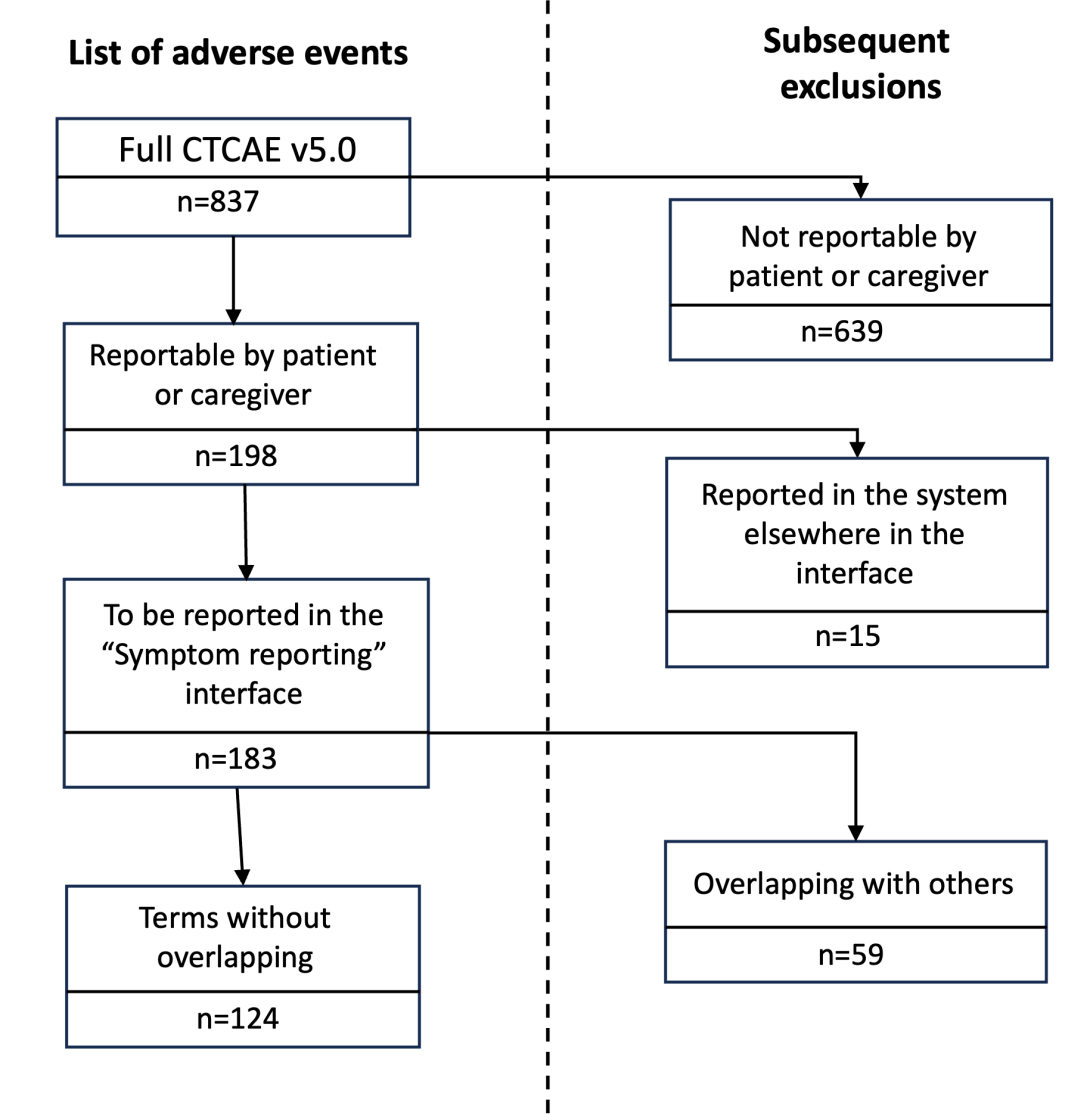
Symptoms selection process. The Common Terminology Criteria for Adverse Events (CTCAE) revision steps and the number of symptoms selected at each step. Overlapping terms mean specialized adverse events that were subsumed by the more general ones.

As a result of the process summarized in Figure 1, we obtained a list of 124 AEs. The detailed symptoms selection process is described and the results of each step are depicted in Figure 2. Forty-nine symptoms are expressed as “present or absent,” and 75 are associated with up to 4 possible “description levels.”

Table 2 provides an excerpt of the developed terminology, highlighting (1) the differences between the original CTCAE terms and their corresponding translations into lay language (column “CAPABLE term”), (2) the Systematized Nomenclature of Medicine code associated with each AE, (3) the levels that are shown to the patients in their app in order to grade the severity of the symptom (columns “Level 1” to “Level 4”), and (4) the mapping between the patient description levels and the original CTCAE grades (last column).

**Table 2.** A snapshot of the developed terminology.

CTCAE^a^ term	CAPABLE^b^ term	SNOMED^c^ code	Level 1	Level 2	Level 3	Level 4	Map to CTCAE grade
Palmar-plantar erythrodysesthesia syndrome	Hand-foot syndrome (rash of the hands or feet that can cause cracking, peeling, redness, or pain)	403638003	Condition without pain that does not interfere or that interferes a little bit with my usual or daily activities (mild)	Condition with pain that interferes somewhat with my usual or daily activities (moderate)	Condition with pain that interferes quite a bit with my usual or daily activities (severe)	Condition with pain that interferes with my daily self-care activities (very severe)	1→1 2, 3 → 2 4 → 3
Skin hypopigmentation	Patches of skin that are lighter than your overall skin tone	23006000	Skin hypopigmentation with no psychosocial impact	Skin hypopigmentation with associated psychosocial impact	—^d^	—	1 → 1 2 → 2
Anosmia	Lost or changed sense of smell	44169009	Present	—	—	—	1 → 1
Anorexia	Decreased appetite	81492003	Decrease of appetite, but I eat or drink almost as usual (mild)	I cannot eat or drink as usual, but I have not lost weight (moderate)	I cannot eat or drink as usual, and I have lost weight (severe)	—	1 → 1 2 → 2 3 → 3
Diarrhea	Diarrhea (loose or watery stools)	62315008	Increase of <4 stools per day compared to usual amount of stools per day	Increase of 4‐6 stools per day compared to usual amount of stools per day	Increase of ≥7 stools per day compared to usual amount of stools per day	—	1 → 1 2 → 2 3 → 3
Amenorrhea	Absence of menstrual periods for 3 consecutive menstrual cycles	14302001	Present	—	—	—	1 → 2

a CTCAE: Common Terminology Criteria for Adverse Events.

b CAPABLE: Cancer Patients Better Life Experience.

c SNOMED: Systematized Nomenclature of Medicine.

d Not applicable.

The mapping between the severity levels and the corresponding CTCAE grades is stored in the CAPABLE DSS and enables the conversion of the patient’s selection into a CTCAE value, which is necessary for executing the guidelines. In addition, each CTCAE grade is mapped to the corresponding Systematized Nomenclature of Medicine–Clinical Terms code for interoperability purposes. The information regarding the symptoms and their severity is stored in the CAPABLE database, which is modeled according to the Observational Medical Outcomes Partnership common data model.

### Implementation of the CAPABLE Terminology

**Figure 3.**
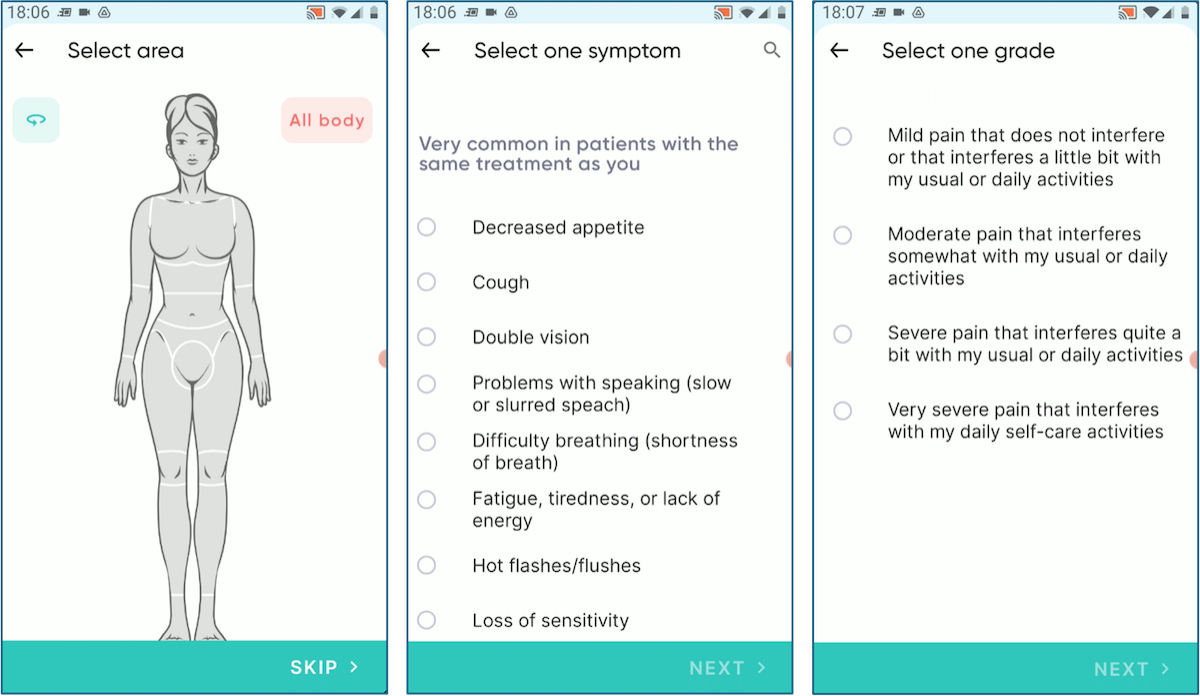
Symptom reporting in the CAPABLE (Cancer Patients Better Life Experience) patient app. From left to right: selecting the affected body part, list of symptoms (if a body part is selected, a filter is applied to the list), and description levels for the pain adverse event.

Figure 3 depicts the integration of the proposed terminology within the user interface of the CAPABLE app for patients and their informal caregivers.

Within the physicians’ dashboard, the CTCAE was implemented in its original form, without modifications, being a terminology specifically developed for clinical use (see the *Methods* section).

**Figure 4.**
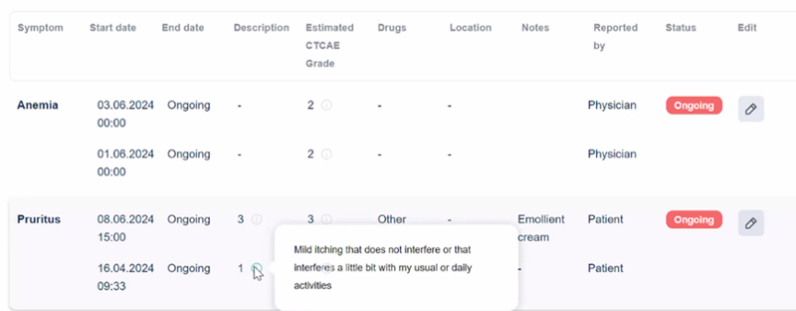
The CAPABLE (Cancer Patients Better Life Experience) physicians’ dashboard. The graphical user interface dedicated to health care providers shows symptoms entered by patients, together with their severity level and the mapped Common Terminology Criteria for Adverse Events grade and those entered by doctors.

Patients eligible for home monitoring with the CAPABLE system are supposed to periodically go to the hospital for control visits. In between visits, doctors can monitor patients through the interface illustrated in Figure 4. As shown, they can see both the description level entered by the patient (“Description”) and the corresponding CTCAE level (“Estimated CTCAE Grade”), in addition to the affected body part, if indicated by the patient (“Location”). During the visit, health care providers can update symptoms previously reported by patients (they also can change the level if needed) or add new ones. To this end, they have the full CTCAE terminology available in their graphical user interface. In Figure 4, pruritus has been entered by the patient, while anemia, which is a medical diagnosis, has been entered by the health care provider (“Reported By”).

### Evaluation Results

#### Use of the Symptom Reporting Functionality

During the evaluation, both qualitative and quantitative assessments were conducted. Among the qualitative evaluations, the focus group, held near the conclusion of the study, revealed that patients considered the app easy to use and effective and expressed disappointment about the service coming to an end [37,38].

After the end of the study, all patients were asked to score from 1 to 5 the level of ease of use of the key functionalities of the CAPABLE system, and 36 (80%) of the 45 patients who filled in the usability questionnaire at the end of the study scored it very easy or easy to use. The level of usefulness, rated with the same scoring system, also received positive feedback from patients: noticeably, the highest rated functionality among those available in the app was the symptom reporting, as the patients perceived this collected information as key for the management of their disease during the treatment phase. A total of 11 health care professionals involved in the studies also assessed the level of ease of the patient app. The symptom reporting functionality was rated among the easiest and most useful tasks, together with the home functionalities and the introductory screens.

With respect to the quantitative results, the app was well exploited by the patients enrolled in the clinical pilot studies. In the Italian cohort, 51 (91%) of 56 patients entered at least 1 distinct symptom. In the Dutch cohort, this occurred for 18 (60%) of 30 patients, but it must be considered that 2 patients died because of rapidly progressive disease during the trial. The average number of new symptoms entered per patient each month is 2.1. Furthermore, after the initial entry, patients regularly provided updates on their symptoms.

To assess whether CAPABLE helped patients to report their symptoms, we analyzed data from patients with mRCC, as symptom information was also collected for the control cohort in this group. For the control patients, symptoms were collected only during control visits by health care providers. As illustrated in Table 3, the symptom variety was lower in the control group, likely due to recall bias and to physicians’ tendency to overlook symptoms not perceived as related to cancer treatment. The higher incidence of fatigue in the control cohort can be explained by the fact that CAPABLE implemented a set of interventions, such as motivating patients to perform physical activity, able to mitigate that symptom.

**Table 3.** Symptoms reported in the 2 cohorts^a^.

Adverse event	Occurrence, n
Control cohort	
* Fatigue^b^*	28
* Mucositis*	9
* Palmar-plantar syndrome*	7
* Diarrhea*	4
* Nausea*	3
CAPABLE^c^ cohort	
* Diarrhea*	8
Muscle pain	5
Headache	5
* Fever*	5
Cough	5
* Nausea*	4
* Fatigue*	4
* Mucositis*	4
* Palmar-plantar syndrome*	3
Passing flatus	3
Backache	3

a Occurrences of symptoms entered by at least 3 patients were considered.

b Symptoms appearing in both cohorts are in italics.

c CAPABLE: Cancer Patients Better Life Experience.

#### Issues Overcome by the Novel Terminology

One of the issues that emerged from the initial analysis of the PRO-CTCAE terminology was that there were more AEs than those considered in the PRO-CTCAE that could be reported by patients or their caregivers. During the CAPABLE study, the patients were able to report 24 distinct symptoms that are not mentioned in the PRO-CTCAE, namely: *hand-foot syndrome, back pain, bone pain, burning sensation of skin, cramps, difficulty in walking, diplopia, dysarthria, edema of entire limb, neck pain, pain in limb, pain in the mouth, peripheral sensory neuropathy, red eye, thirst, malaise, influenza-like illness, toothache, hypersomnia, fever, edema of face, pain in face, tremor,* and *limitation of joint movement*. Note that PRO-CTCAE also includes “pain” but without the body site specification, which clinicians consider important. Thus, we believe that, also thanks to the friendly symptom descriptions and search strategy provided in the app, patients (possibly with their caregiver’s help) were able to recognize and report a greater variety of AEs. The “Other” option for symptom reporting has been used only 1 time, for reporting jaundice, which is currently not present either in the CTCAE or in the PRO-CTCAE. This suggests that patients managed most of the time to find the symptoms they were looking for, but also that such a term could be considered an additional term in an updated version of the terminology.

A second issue identified with the PRO-CTCAE was the absence of a direct mapping to CTCAE grades. This would prevent triggering those guidelines recommendations that consider CTCAE grades in their premise. To validate our terminology with respect to this issue, we counted how many different recommendations of that type have been fired during the pilot studies, and that would not be fired in case symptoms were reported in other ways. We found 11 distinct recommendations (related to diarrhea, fatigue, and skin problems) that physicians received, from one to several times, to treat 24 different patients. Without a proper mapping between the symptom reported by the patient and the CTCAE grade, it would not have been possible for the DSS to deliver such recommendations. To assess the level of satisfaction with the triggered recommendations, we collected impressions by 11 health care professionals about the ease and usefulness of the guideline access functionality, on a 1‐5 Likert scale. Regarding ease of use, 7 (60%) of the participants judged the functionality easy to use, while 2 (20%) were neutral, with no participants giving negative feedback on the question. When asked about the usefulness, 4 (40%) of the health care professionals judged the functionality useful, 3 (30%) were neutral, while only 1 health care provider judged it not useful.

A third issue relates to questionnaires designed for administration at fixed intervals, such as on a weekly basis. For example, the PRO-CTCAE instructs: “For each question, please select the one response that best describes your experiences over the past 7 days.*”* This format is not suitable for a monitoring app, where symptoms need to be reported immediately upon occurrence. To support our statement, even if anecdotally, we illustrate the experience, within the CAPABLE study, of a patient who received early diagnosis of a life-threatening condition, thanks to this on-demand symptom reporting feature. In addition to cancer, the patient suffered from a psychiatric disorder for which he was being treated with medications. Through the dashboard, the oncologist noticed, for a few consecutive days, that the home caregiver reported dizziness and falls while walking. The caregiver annotated that this was probably due to the ongoing psychiatric treatment. However, for oncologists, those specific symptoms raised suspicion of brain metastases. Thus, the patient underwent an urgent brain computed tomography scan with contrast. Despite, unfortunately, the presence of metastasis being confirmed, the patient received a prompt diagnosis and started early treatment, also avoiding hospitalization that would have been necessary in case of late diagnosis.

## Discussion

In this paper, we described the terminology adopted in the CAPABLE project for symptom reporting and its integration in graphical user interfaces. Both the terminology and the interfaces were designed by involving patients as the main actors of the requirement analysis, following an iterative codesign process including interviews where we were showing them, one after the other, the graphical user interface mockups [39]. As a result, although the CAPABLE system shares certain features with other systems described in the related work section, it also incorporates distinctive elements that position it beyond the current state of the art.

First, the CAPABLE does not propose a list of AEs that is filtered on the basis of the pathology and treatment. While this kind of filtering, used for example in [15,19,20,22], could represent a way to personalize the user interfaces, it could prevent entering AEs that are unexpected, for example, because the treatment has been introduced recently, and not enough data are available to exclude the possibility of new, still unknown AEs. Instead of shortening the AE list, to facilitate the AE selection for the patient, we use runtime strategies such as showing the most probable AEs at the top of the list (personalized according to the type of cancer and treatment), or filtering according to the body part selected through the graphical interface (Figure 2) [40]. This strategy makes our interface terminology generalizable and usable for different diseases.

Second, the CAPABLE allows a patient to enter symptoms as soon as they manifest, possibly even more frequently than once a day. This is different from the solutions currently available on the market [41] and in the literature [34,42-44] that usually require the patient to periodically fill out a questionnaire. In the CAPABLE paradigm, a patient just reports a symptom when it arises, choosing from different input modalities. This is useful when symptom management requires a timely intervention. On the other hand, if the patient does not experience any symptoms, he or she is not required to interact with the system. Questionnaires are also used in CAPABLE but just to collect data about psychological and nutritional status, with a frequency that is personalized according to the last entered data (the frequency of administration increases if critical values are reached in previous answers). The questionnaires were selected by the medical experts of the CAPABLE team to address the conditions that require more details than a single AE item might allow. The patient also receives a reminder in case any data are entered for more than a predefined period.

Finally, defining an extended interface terminology starting from the CTCAE can be beneficial for patients, giving them the potential to gradually learn to evaluate their own health condition more objectively. This learning process is also facilitated by the app, which provides timely and focused educational material at symptom entry: for each symptom, an info button provides an exhaustive description of the symptom itself.

Overall, the results show the efficacy of our design choices in collecting high-quality and timely data from home patients. However, not all patients used the app in the same way. The app was deployed in 2 countries with different patient populations and organizational settings. Italian patients showed greater engagement with the app compared to Dutch patients, likely due to the more consistent technical support provided in Italy, where a dedicated bioengineering team was available. This difference was observed in overall app usage and was also reflected in the use of the symptom reporting feature. This highlights the importance of closely monitoring patients and promptly addressing their issues during the telemonitoring period.

As another limitation, it is important to point out that the proposed CAPABLE terminology is still lacking an external validation phase. As a matter of fact, the symptom reporting functionality was indirectly assessed through system usage during the CAPABLE clinical study, giving the encouraging results described above. On the other hand, we did not implement a specific post-deployment evaluation strategy specifically targeted to the developed terminology. As a future activity, a formal validation involving external stakeholders and other patient groups will be planned.

As a final consideration, we remark that it is hard to separate the development of a terminology from its intended use. The CAPABLE terminology is intended to be used by patients and their caregivers in mHealth apps dedicated to home patients’ monitoring, and this has influenced some design choices. However, the obtained instrument is general enough to be exploited by different similar mHealth apps.

## Supplementary material

10.2196/65504 Multimedia Appendix 1 The CAPABLE (Cancer Patients Better Life Experience) terminology, including the 124 selected adverse events, with the corresponding Common Terminology Criteria for Adverse Events (CTCAE) terms, patient-oriented display of the term, description levels, and description of each level to the CTCAE grades.

## Abbreviations

ADL: activity of daily living
AE: adverse event
CAPABLE: Cancer Patients Better Life Experience
CTCAE: Common Terminology Criteria for Adverse Events
DSS: decision support system
ePROMs: electronic patient-reported outcomes measures
ICI: immune checkpoint inhibitor
mHealth: mobile health
mRCC: metastatic renal cell carcinoma
NCI: National Cancer Institute
PRO: patient-reported outcome
QoL: quality of life
STAR: Symptom Tracking and Reporting
